# Female breast cancer trends: A South African perspective

**DOI:** 10.4102/sajr.v29i1.3117

**Published:** 2025-05-31

**Authors:** Heliodora De Lima, Sofia Ramos, Leisha Rajkumar, Herbert Cubasch

**Affiliations:** 1 Department of Diagnostic Radiology, Faculty of Health Sciences, University of the Witwatersrand, Johannesburg, South Africa; 2 Department of Surgery, Faculty of Health Sciences, University of the Witwatersrand, Johannesburg, South Africa

**Keywords:** breast cancer, age, demographic characteristics, tumour characteristics, breast cancer trends, breast imaging unit

## Abstract

**Background:**

Some clinicians and radiologists in South Africa (SA) suspect that aggressive subtypes of breast cancer are becoming more prevalent and that patients are presenting at younger ages.

**Objectives:**

This study aimed to analyse the prevalence and trends in female breast cancer presentations at a Breast Unit in Johannesburg, SA, by comparing data from 2012 and 2022.

**Method:**

A retrospective study was conducted at a tertiary hospital in Johannesburg. Records of female patients diagnosed with breast cancer between 2012 and 2022 were analysed. Demographic data, ultrasound or mammography findings, and tumour characteristics were compared.

**Results:**

A total of 493 records were reviewed: 165 (33.5%) from 2012 and 328 (66.5%) from 2022. The mean ± standard deviation (s.d.) age at presentation was 56.8 ± 16.8 years in 2012 and 54.1 ± 13.6 years in 2022 (*p* = 0.056). Tumours were smaller in 2022 (mean ± s.d., 35.0 mm ± 24.0 mm) compared to 2012 (48.1 mm ± 21.5 mm) (*p* < 0.001). A higher proportion of women had positive oestrogen receptor status in 2022 (*p* = 0.005). No differences were observed in molecular subtypes.

**Conclusion:**

No significant change was found in the mean age at presentation, suggesting a stable demographic profile. However, reproductive, hormonal, and lifestyle factors may contribute to the rising prevalence among women aged 40–49 years. Smaller tumours likely reflect increased awareness and clinical breast examinations at local clinics.

**Contribution:**

This single-institution study underscores the need for broader national research to inform breast cancer screening and imaging guidelines.

## Introduction

According to some research, breast cancer is increasingly affecting younger individuals.^1,2,3^ Although this is a valid concern among healthcare professionals, it can be challenging for clinicians, particularly radiologists, to observe trends in breast cancer patients presenting at imaging units or breast clinics. Although it may seem that younger women are being diagnosed more frequently than in the past, this perception requires statistical evidence for confirmation and is an important clinical question to investigate further.

Based on the 2020 Global Cancer Statistics (GLOBOCAN) data, breast cancer is the most diagnosed cancer among women, with approximately 2.3 million new cases reported annually, making up 11.7% of all cancer cases.^4^ It is also the leading cause of cancer-related deaths in women globally.^4^ According to the 2019 South African National Cancer Registry (NCR), breast cancer accounted for 23.2% of all female cancers, with a lifetime risk of 1 in 27^5^ and is the leading cancer in South African women.^6,7,8^ The incidence and death rates of breast cancer have been on the rise because of various factors such as population growth, increased life expectancy, globalisation and higher prevalence of risk factors, as well as improved cancer recording and detection.^4,9,10^ As a result, it is expected that by 2030, the distribution of cancer cases worldwide will increase across all age groups.^6,9^

Although most breast cancer patients are postmenopausal,^3,9^ there has been a growth in premenopausal breast cancer, leading to a younger presentation.^3^ Emerging evidence indicates an upward trend of breast cancer in women under 40.^1,2,3^ The Group of Cancer Epidemiology and Registration in Latin Language Countries (GRELL) study examined European epidemiological data, revealing an annual increase of 1.2% from 1990 to 2008.^2^

Definitions of ‘young women’ with breast cancer vary but generally refer to women who are less than or equal to 40 years old.^11^ Young breast cancer patients often have more aggressive tumours, and a poorer prognosis compared to older patients.^1,3,9,11^ In China and Africa, the age of onset for breast cancer is earlier than in Europe and the United States (US).^11,12^ In South Africa (SA), however, there has been little research dedicated to breast cancer trends in the younger age groups, which might inform local screening strategies. In 2021, The American College of Radiology (ACR) and the Society of Breast Imaging (SBI) recommended annual screening mammography starting at the age of 40 years for women with average risk.^13,14^ Initiating screening at this age has been shown to reduce mortality rates significantly, enable early detection and diagnosis, and provide more effective treatment options.^13,14^ The Radiological Society of South Africa (RSSA) and Breast Imaging Society of South Africa (BISSA) also recommend annual screening mammography and regular breast examinations starting at the age of 40 years,^13^ which aligns with the ACR and SBI guidelines. The SA National Department of Health (DoH) has established guidelines for breast cancer screening and detection to address the diverse risk profiles of women in the country.^15^ However, because of resource constraints, the public healthcare system in SA offers less robust mammography screening services^15^ compared to patients with access to private healthcare that benefit from more reliable and effective screening services.^13^

The high prevalence of human immunodeficiency virus (HIV) in SA may lead to the coexistence of HIV positivity and breast carcinoma.^16^ However, studies have shown no significant causal link between the two.^16^ A study conducted by Cubasch et al. showed that HIV-positive women with breast cancer were younger than HIV-negative women, however, tumour characteristics, stage and grade were not significantly influenced by HIV status.^16,17^

Compared to other developed nations, less is known in SA about the risk factors, clinical and histological characteristics, gene expression and molecular patterns among breast cancer patients.^12,18,19^ Given the significant disease burden, conducting ongoing research to address uncertainties, explore relationships and better understand its prevalence is essential. Therefore, further research on the age of first breast cancer presentation and its characteristics in the South African population is necessary, as this knowledge can help improve care and potentially reduce the prevalence and mortality in SA.

This study assessed the prevalence and compared the demographic and tumour characteristic trends of female breast cancer at Helen Joseph Hospital (HJH) Breast Imaging Unit (BIU), a tertiary public hospital in Johannesburg, SA.

## Research methods and design

The HJH Breast Clinic is one of the few specialised public breast clinics in Gauteng, SA that provides open access to all patients needing breast examinations and investigations. The clinic diagnoses and treats over 300 new breast cancer cases per year.

In this retrospective analysis within the study periods (2012 and 2022) at the BIU of HJH, females aged 18 years and older with histologically verified breast cancer and/or with a second primary breast cancer diagnosed in these years, were included. Patients with recurrent breast cancer of the same histological type, breast sarcomas and those undergoing follow-up were excluded.

Demographic variables such as the age at breast cancer diagnosis and the HIV status were collected. The imaging tumour characteristics included the size of the primary tumour (the largest dimension measured on either breast ultrasound or mammography) and suspicious microcalcifications. The recorded histological tumour characteristics consisted of the morphological tumour type (ductal, lobular, mixed or other), nuclear grade (1, 2 or 3), immunohistochemistry (tumour receptor status), Ki-67 percentage and the breast cancer subtypes (Luminal A, Luminal B, human epidermal growth factor receptor 2 [HER2]-enriched or triple-negative breast cancer [TNBC]).

The morphological tumour types were categorised into invasive ductal carcinoma, invasive lobular carcinoma, mixed category (ductal and invasive lobular carcinoma combined) and other (mucinous, invasive papillary, micropapillary, metaplastic and secretory carcinomas). The Allred scoring system quantified the oestrogen receptor (ER) and progesterone receptor (PR) status. A total score of less than or equal to 2 was considered negative, while a score greater than or equal to 3 was classified as positive. Interpretation of the HER2 status considered a tumour staining intensity of 3+ or a positive FISH test result as positive. A staining of 0, 1+ or 2+ (equivocal) and an absent FISH result were recorded as HER2 negative. Breast cancer subtypes were classified as Luminal A (ER+ and/or PR+, HER2-), Luminal B (ER+ and/or PR+, HER2+), HER2-enriched (HER2+, ER-, PR-) and TNBC (ER-, PR-, HER2-). As Ki-67 was unavailable in the 2012 data set, nuclear grade, HER2 and FISH results were used to classify the Luminal cancers.

The data from patients’ files was entered into a Microsoft Excel spreadsheet. Categorical variables were presented as frequency and percentages, while continuous variables were presented as mean ± standard deviation (s.d.) or median and interquartile range (IQR) if not normally distributed. A bar graph was used to demonstrate the differences between the molecular subtypes. Continuous variables were compared between the two years using the Student’s *t*-test. Categorical variables were compared between the two years using Pearson’s Chi-square test and Fisher’s exact test, where data were skewed. A two-sided *p*-value <0.05 was considered statistically significant throughout. Analysis was performed using Stata Version 18.

### Ethical considerations

The study was approved by the Human Research Ethics Committee (HREC) of the University of the Witwatersrand (reference no.: M230324).

## Results

### Demographic characteristics of the study population

Of all the women seen in the BIU over the 2 years, 493 records were selected for inclusion and made up the study sample. In 2012 165 (33.5%) records were evaluated, compared to 328 records (66.5%) in 2022.

Table 1 describes and compares the demographic characteristics of the study population. The mean age ± s.d. was similar between the two years (*p* = 0.056). There was a statistically significant difference in the age distribution between the years, *p* = 0.013. The prevalence of women with breast cancer aged 40–49 years in 2012 was 19.4% versus 26.8% in 2022. In women aged ≥ 70 years, the prevalence was 26.1% in 2012 versus 14.3% in 2022. The prevalence of HIV-positive individuals was similar (*p* = 0.962).

**TABLE 1:** Demographic characteristics of women presenting with breast cancer at Helen Joseph Hospital Breast Imaging Unit in 2012 and 2022.

Variable	Total				2012				2022				*p*
	*n*	%	Mean	s.d.	*n*	%	Mean	s.d.	*n*	%	Mean	s.d.	
Number of records	493	100.0	-	-	165	33.5	-	-	328	66.5	-	-	-
**Age at diagnosis in years**	-	-	55.0	14.7	-	-	56.8	16.8	-	-	54.1	13.6	0.056
**Age at diagnosis in years**	-	-	-	-	-	-	-	-	-	-	-	-	0.013
< 40	76	15.4	-	-	28	16.9	-	-	48	14.6	-	-	-
40–49	120	24.3	-	-	32	19.4	-	-	88	26.8	-	-	-
50–59	114	23.1	-	-	36	21.8	-	-	78	23.8	-	-	-
60–69	93	18.9	-	-	26	15.8	-	-	67	20.4	-	-	-
≥ 70	90	18.3	-	-	43	26.1	-	-	47	14.3	-	-	-
**HIV status**	-	-	-	-	-	-	-	-	-	-	-	-	0.962
Negative	254	77.0	-	-	62	76.0	-	-	192	77.0	-	-	-
Positive	77	23.0	-	-	19	24.0	-	-	58	23.0	-	-	-

Note: Row percentages shown. Total missing data: HIV (*n* = 162).

s.d., standard deviation.

### Tumour characteristics of the study population

**FIGURE 1:**
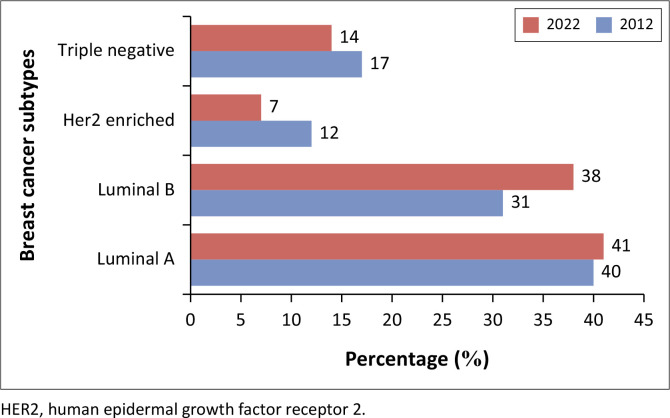
Distribution of molecular subtypes of women presenting with breast cancer at Helen Joseph Hospital Breast Imaging Unit in 2012 and 2022.

The tumour characteristics are presented in Table 2. In 2022, the mean ± s.d. tumour size reported on imaging was 35.0 mm ± 24.0 mm, whereas, in 2012, it was 48.1 mm ± 21.5 mm, *p* < 0.001. A significantly higher percentage of women had a positive ER status in 2022 compared to 2012 (78.0% vs. 65.5%, *p* = 0.005). Despite the latter, however, the molecular subtype did not differ, and for both years, Luminal A was the most common (39.6% in 2012 vs. 40.9% in 2022, *p* = 0.287), as demonstrated in Figure 1.

**TABLE 2:** Tumour characteristics and radiological features of women presenting with breast cancer at Helen Joseph Hospital Breast Imaging Unit in 2012 and 2022.

Variable	Total				2012				2022				*p*
	*n*	%	Mean	s.d.	*n*	%	Mean	s.d.	*n*	%	Mean	s.d.	
**Tumour size (mm)**	-	-	37.7	23.4	-	-	48.1	21.5	-	-	35.0	24.0	< 0.001
**Suspicious microcalcifications**	-	-	-	-	-	-	-	-	-	-	-	-	0.091
Absent	201	44.0	-	-	57	39.0	-	-	144	47.0	-	-	-
Present	251	56.0	-	-	90	61.0	-	-	161	53.0	-	-	-
**Morphological type**	-	-	-	-	-	-	-	-	-	-	-	-	0.174
Ductal	446	90.0	-	-	150	90.0	-	-	296	90.0	-	-	-
Lobular	25	5.0	-	-	11	6.0	-	-	14	4.0	-	-	-
Mixed	1	1.0	-	-	1	1.0	-	-	0	0.0	-	-	-
Other	23	4.0	-	-	5	3.0	-	-	18	6.0	-	-	-
**Nuclear grade**	-	-	-	-	-	-	-	-	-	-	-	-	0.909
1	35	7.0	-	-	10	6.0	-	-	25	8.0	-	-	-
2	251	52.0	-	-	83	53.0	-	-	168	52.0	-	-	-
3	197	41.0	-	-	65	41.0	-	-	132	40.0	-	-	-
**ER status**	-	-	-	-	-	-	-	-	-	-	-	-	0.005
Negative	120	25.8	-	-	48	34.5	-	-	72	22.0	-	-	-
Positive	346	74.2	-	-	91	65.5	-	-	255	78.0	-	-	-
**PR status**	-	-	-	-	-	-	-	-	-	-	-	-	0.740
Negative	207	44.8	-	-	63	46.0	-	-	144	44.3	-	-	-
Positive	255	55.2	-	-	74	54.0	-	-	181	55.7	-	-	-
**HER2 status**	-	-	-	-	-	-	-	-	-	-	-	-	0.584
Negative	326	71.3	-	-	98	73.1	-	-	228	70.6	-	-	-
Positive	131	28.7	-	-	36	26.9	-	-	95	29.4	-	-	-
**Molecular subtype**	-	-	-	-	-	-	-	-	-	-	-	-	0.287
Luminal A	185	40.5	-	-	53	39.6	-	-	132	40.9	-	-	-
Luminal B	163	35.7	-	-	42	31.3	-	-	121	37.5	-	-	-
HER2 enriched	40	8.8	-	-	16	11.9	-	-	24	7.4	-	-	-
Triple negative	69	15.0	-	-	23	17.2	-	-	46	14.2	-	-	-

Note: Row percentages shown. Total missing data: tumour size (*n* = 21), suspicious microcalcifications (*n* = 43), nuclear grade (*n* = 9), ER status (*n* = 27), PR status (*n* = 31), HER2 status (*n* = 36) and molecular subtype (*n* = 36).

mm, millimetres; s.d., standard deviation; ER, oestrogen receptor; PR, progesterone receptor; HER2, human epidermal growth factor receptor 2.

Table 3 shows the distribution of molecular subtypes across age categories. Overall, Luminal A was the most prevalent breast cancer subtype in women aged 70 years and older (59.8%). In contrast, Luminal B was the most common subtype of breast cancer in women under 40 years (57.1%). A similar trend to the combined total data were shown in 2012 and 2022. Triple-negative breast cancer was most prevalent among patients aged 50–59 years within the study population (25.0%). The proportion of patients with TNBC in the two comparative years was comparable to the total, with 32.3% in 2012 and 22.1% in 2022. The proportion of patients aged 50–59 years with HER2 enriched was 25.8% in 2012 and 5.2% in 2022.

**TABLE 3:** Distribution of molecular subtypes by age categories of women presenting with breast cancer at Helen Joseph Hospital Breast Imaging Unit in 2012 and 2022.

Age categories in years	Total								2012								2022							
	Luminal A		Luminal B		HER2 enriched		Triple negative		Luminal A		Luminal B		HER2 enriched		Triple negative		Luminal A		Luminal B		HER2 enriched		Triple negative	
	*n*	%	*n*	%	*n*	%	*n*	%	*n*	%	*n*	%	*n*	%	*n*	%	*n*	%	*n*	%	*n*	%	*n*	%
< 40	21	30.0	40	57.1	3	4.3	6	8.6	8	36.4	12	54.6	0	0.0	2	9.1	13	27.1	28	58.3	3	6.3	4	8.3
40–49	44	39.3	43	38.4	8	7.1	17	15.2	8	32.0	10	40.0	2	8.0	5	20.0	36	41.4	33	37.9	6	6.9	12	13.8
50–59	35	32.4	34	31.5	12	11.1	27	25.0	8	25.8	5	16.1	8	25.8	10	32.3	27	35.1	29	37.7	4	5.2	17	22.1
60–69	36	42.4	27	31.8	10	11.8	12	14.1	9	45.0	5	25.0	2	10.0	4	20.0	27	41.5	22	33.9	8	12.3	8	12.3
≥ 70	49	59.8	19	23.2	7	8.5	7	8.5	20	55.6	10	27.8	4	11.1	2	5.6	29	63.0	9	19.6	3	6.5	5	10.9

Note: Row percentages shown.

HER2, human epidermal growth factor receptor 2.

## Discussion

This study assessed the prevalence and comparisons between the demographic and tumour characteristic trends of 493 women diagnosed with breast cancer, 165 (33.5%) of which were recorded in 2012 and 328 (66.5%) in 2022. There was no change in the mean ± s.d. age of breast cancer presentation, in 2012 it was 56.8 ± 16.8 and in 2022 it was 54.1 ± 13.6 (*p* = 0.056). The tumours were smaller in 2022 (mean ± s.d., 35.0 mm ± 24.0 mm) than in 2012 (48.1 mm ± 21.5 mm), *p* < 0.001 and a higher percentage of women had a positive ER status in 2022 compared to 2012 (*p* = 0.005).

The number of reviewed records doubled in 2022 (66.5%) compared to 2012 (33.5%). This could be because of increased awareness of breast cancer, leading more patients to present to the breast clinic and possibly more external referrals. Additionally, access to the older records was hampered by poor record keeping and administration, making the records unretrievable in 2012, which could also have contributed to the fewer records that year. A new Picture Archiving and Communication System (PACS) was also introduced, which led to a lack of continuity.

This study examined the trend differences between population groups in 2012 and 2022. The authors recognised the importance of understanding the latest trends within the 2022 data subset and compared the study’s findings to several local and international studies. These comparative references are integrated within the text and are further summarised in Table 4.

**TABLE 4:** Comparative analysis of the literature with other case series of breast cancer.

Study	Current study	Cubasch^17^	McCormack^18^	Sinha^12^	Ayeni^20^	Ohene^25^	Rahman^24^	Brinton^34^
Year	2022	2006–2012	2006–2012	2016–2017	2015–2019	2004–2009	2003–2008	1992–2004
Site	SA	SA	SA	SA	SA	Ghana	Nigeria	US
Number of patients	328	1092	1247	475	2367	330	82	387 231
**Age in years**								
Median	53.0	-	-	54	55.1	-	-	-
IQR	-	-	-	44–66	44.8–65.8	-	-	-
Mean	54.1	-	55.3	-	-	49.1	48.98	-
s.d.	13.6	-	14.3	-	-	15.3	10.97	-
**Age categories in years**								
< 40								
*n*	48	134	182	-	326	-	16	-
%	14.6	17.5	15.0	-	13.8	-	19.5	-
40–49								
*n*	88	201	290	-	568	-	26	-
%	26.8	26.3	23.9	-	24.0	-	31.70	-
50–59								
*n*	78	198	310	-	557	-	22	-
%	23.8	25.9	25.5	-	23.5	-	26.80	-
60–69								
*n*	67	127	221	-	499	-	14	-
%	20.4	16.6	18.2	-	20.1	-	17.1	-
≥ 70								
*n*	47	105	213	-	417	-	4	-
%	14.3	13.7	17.5	-	17.6	-	4.9	-
**HIV positive**								
*n*	58	151	153	50	499	-	-	-
%	23.0	19.7	18.2	10.5	21.1	-	-	-
**Tumour size**								
Size (mm)	-	-	-	-	-	-	21–50	≤ 20
Mean ± s.d. (mm)	35 ± 24	-	-	-	-	-	-	-
**Patients with specified tumour size**								
*n*	-	-	-	-	-	-	50	209 203
%	-	-	-	-	-	-	61.0	54.0
**Most frequent nuclear grade**								
Low grade								
*n*	-	-	-	-	-	-	-	182 928
%	-	-	-	-	-	-	-	47.2
Grade 2								
*n*	168	409	455	-	1119	-	-	-
%	52.0	46.6	46.8	-	55.1	-	-	-
Grade 3								
*n*	-	-	-	-	-	145	-	-
%	-	-	-	-	-	53.7	-	-
**ER status**								
ER negative								
*n*	72	352	376	-	-	36	-	61 434
%	22.0	36.4	35.0	-	-	53.0	-	15.9
ER positive								
*n*	255	614	696	-	-	32	-	208 616
%	78.0	63.6	64.9	-	-	47.0	-	53.9
**PR status**								
PR negative								
*n*	144	462	500	-	-	59	-	-
%	44.3	48.0	46.9	-	-	86.8	-	-
PR positive								
*n*	181	499	567	-	-	9	-	-
%	55.7	51.9	53.0	-	-	13.2	-	-
**HER2 status**								
HER2 negative								
*n*	228	682	762	-	-	43	-	-
%	70.6	73.6	74.0	-	-	79.6	-	-
HER2 positive								
*n*	95	245	267	-	-	11	-	-
%	29.4	26.4	26.0	-	-	20.4	-	-
**Luminal A**								
*n*	132	-	551	276	1434	-	-	-
%	40.9	-	53.7	69.70	60.6	-	-	-
**Luminal B**								
*n*	121	-	150	49	410	-	-	-
%	37.5	-	14.6	12.37	17.3	-	-	-
**HER2 enriched**								
*n*	24	-	117	26	148	-	-	-
%	7.4	-	11.0	6.57	6.3	-	-	-
**Triple negative**							Most common	
*n*	46	196	209	45	375	23	-	-
%	14.2	20.7	20.4	11.36	15.8	42.7	-	-

Note: Please see full reference list of this article, De Lima H, Ramos S, Rajkumar L, Cubasch H. Female breast cancer trends: A South African perspective. S Afr J Rad. 2025;29(1), a3117. https://doi.org/10.4102/sajr.v29i1.3117, for more information.

s.d., standard deviation; IQR, interquartile range; mm, millimetres; cm, centimetres; ER, oestrogen receptor; PR, progesterone receptor; HER2, human epidermal growth factor receptor 2; US, United States.

This study found the mean age ± s.d. to be similar between the two years (56.8 ± 16.8 years in 2012 vs. 54.1 ± 13.6 years in 2022, *p* = 0.056), consistent with another study^18^ and similar to the median age reported in other studies.^12,20^ This consistency indicates that the demographic profile and risk factors of breast cancer patients have remained stable over time, as demonstrated by similar findings across various studies conducted in SA and within the hospital where this study took place. The younger age structure of the South African population, which includes fewer older women, and genetic and environmental risk factors, may explain the lower mean age at diagnosis compared to the older population profiles in the US and Europe.^11,12^

Although the mean age in this study was comparable, there were some significant differences in the categorical age distribution (*p* = 0.013) between 2012 and 2022. The percentage of women in the 40–49 years age range who received a breast cancer diagnosis rose from 19.4% in 2012 to 26.8% in 2022. Similar to findings in Brazil, there has been a consistent rise in hospital admissions for female breast cancer patients in this age category.^21^ The increasing prevalence of breast cancer among women aged 40–49 years observed in this study may be attributed to a combination of reproductive and hormonal factors, as well as alterations in lifestyle.^6,21,22,23^ Reproductive and hormonal factors such as early menarche, use of oral hormonal contraception, late first pregnancy age (after 30 years), nulliparity or fewer gestations, and reduced breastfeeding could be the reasons to support a higher prevalence in these women.^6,21,22,23^ It is plausible that lifestyle factors, such as increased sedentary behaviour, obesity and higher alcohol consumption, may also play a role in the elevated prevalence of breast cancer in this age group.^6,21,22,23^ Given that most diagnoses in this study occurred in women aged 40–49 years, which is consistent with similar studies conducted in sub-Saharan Africa (SSA),^17,20,24,25^ it is important to emphasise that screening of this age group should be encouraged, especially in the SA context.

This study found that the prevalence of breast cancer in women 40 years and younger did not increase from 2012 to 2022; instead, it slightly decreased (16.9% in 2012 vs. 14.6% in 2022). There was also a difference between the 60–69 years age group (15.8% in 2012 vs. 20.4% in 2022) and the 70-year-old and older subgroup categories (26.1% in 2012 vs. 14.3% in 2022). The women 70 years and older presented less in 2022, which may be explained by barriers to care affecting the elderly, as they were less likely to present to the hospital during the coronavirus disease 2019 (COVID-19) pandemic. Soyder, et al. found that the elderly population, particularly those aged 65 years and older, faced significant barriers to accessing care during the pandemic, which included fear of COVID-19 transmission, high mortality rates because of comorbidities and restrictions on movement.^26^ The reduced prevalence in individuals aged 70 years and older in 2022, compared to 2012, may also be explained by the lack of encouragement for screening in this age group, as there is ongoing debate about whether screening should continue beyond the age of 70 years.^27^

In this study, the recorded HIV status did not indicate a change over the 10 years. The prevalence of HIV among breast cancer patients was approximately 23.0% in 2022 and 24.0% in 2012 (*p* = 0.962). Other studies reported similar prevalence results for HIV-positive patients who were newly diagnosed with breast cancer in SA.^17,18,20^ The unchanged prevalence of HIV-positive results may be linked to HIV campaigns, suggesting that they may contribute to improving knowledge about HIV and reducing its stigma.^28^ Studies have shown that women with a dual diagnosis of HIV and breast cancer experience significantly higher mortality and morbidity rates compared to HIV-uninfected breast cancer patients.^20,29^ Additionally, women with HIV tend to be diagnosed with breast cancer at a younger age.^17^ HIV infection can affect the effectiveness of breast cancer treatments, and these women are less likely to achieve a pathologically complete response after neoadjuvant chemotherapy compared to those without HIV.^30^ Incomplete treatment may also increase the risk of recurrence and ultimately result in lower survival rates.^29^

In 2022, the mean ± s.d. tumour size reported on imaging was 35 mm ± 24 mm, compared to 48.1 mm ± 21.5 mm in 2012 (*p* < 0.001), indicating that tumours were significantly smaller in 2022. Despite the impact of COVID-19, this trend could be attributed to improved patient health education. Women are identifying tumours earlier, at smaller sizes and lower stages than in previous years, thanks to public health campaigns and awareness programmes that inform them about the risks, signs and symptoms of breast cancer.^31^ The smaller tumour sizes observed in 2022 can likely be attributed to the screening and policy initiatives implemented by the National DoH. These initiatives include raising awareness about breast cancer, promoting breast self-examinations (BSE) and conducting clinical breast examinations (CBE) by qualified healthcare professionals at local clinics, contributing to the earlier detection of smaller tumours.^15^ As a result, women visiting primary healthcare facilities or hospitals for other reasons should be targeted for opportunistic CBE and awareness initiatives. In low-resource areas, BSE plays a vital role in breast health awareness and early detection.^15^ Furthermore, there have been significant advancements in imaging techniques, particularly vacuum-assisted biopsies, stereotactic biopsies, enhanced resolution and improvements in ultrasound imaging.^27^ Detecting breast cancer at a smaller size is advantageous in terms of management, treatment and prognosis.^13,15,32^

The morphological tumour types observed in this study were consistent with those reported in the literature. As documented in previous studies, invasive ductal carcinoma was the most frequently diagnosed type, comprising 90.0% of the total tumours for 2012 and 2022.^9,18,20,25,33,34^ In contrast, lobular carcinoma comprised only 5.0% of the study population.

Not only does the understanding of the hormone receptor status and molecular subtypes describe the biology of breast cancer and influence its clinical behaviour, but it is also essential for determining the most effective treatment and management plans as the treatment strategies differ depending on the molecular subtype.^9,33^ Molecular subtypes such as HER2-enriched and TNBC are often linked to more aggressive forms of the disease.^9,33^ Hormone receptor-positive subtypes, such as Luminal A, have a low grade, are less aggressive, and are associated with a more favourable prognosis.^9,33^ In contrast, Luminal B cancers have a worse prognosis than Luminal A tumours because of the higher histological grade.^9,33^

This study observed a statistically significant difference in ER status between 2012 and 2022, with ER-positive cases increasing from 65.5% to 78.0% (*p* = 0.005). This shift in the hormone receptor may be attributed to changes in pathology reporting and analysis, potentially involving a more rigorous application of the Allred scoring system by histopathologists.^35^ The Allred scoring system, which evaluates both the proportion of positive cells and the intensity of staining, has provided a more sensitive and specific evaluation of ER status compared to conventional methods.^35^ There were no significant differences in the PR and HER2 statuses between 2012 and 2022. Because ER-positive tumours represent the majority, it shows that less aggressive breast cancer was found in this study population, as was also demonstrated elsewhere.^12,17,18,34^ Other studies found a different ratio,^25^ with a higher number of ER-negative tumours reflecting more aggressive tumour biology.^34^ The latter are poorly differentiated and lead to more advanced diseases and poorer prognoses.^3,18^

The distribution of breast cancer subtypes in this study population closely aligns with the proportions reported in a review.^9^ In this study, Luminal molecular subtypes accounted for 76.2% of the total population, while HER2 enriched made up 8.8% and TNBC comprised 15.0%. In comparison, the review indicated that Luminal breast cancers represent approximately 70% of all cases, HER2-enriched accounts for 10%–15% and TNBC makes up about 20% of breast cancers overall.^9^

In contrast to the widely held notion that SSA women are more likely to be diagnosed with more aggressive breast cancer subtypes, especially TNBC,^12,19,24^ this study revealed that Luminal A was the most prevalent molecular subtype in both 2012 (39.6%) and 2022 (40.9%), followed by Luminal B as illustrated in Figure 1. A comparative analysis revealed that the most prevalent subtype of breast cancer in SA was also Luminal A.^12,20,29^ This, again, may be explained by the application of the Allred scoring system.^35^ In addition, a more precise classification of tumour subtypes would have been possible with the use of the Ki-67. The TNBC subgroup in both years was similar in this study (17.2% in 2012 and 14.2% in 2022), in keeping with the findings reported in other local studies.^17,18,20,29^ The current authors did not see a pattern of more aggressive tumours such as HER2 enriched and TNBC subtypes.

Research has found an association between the patient’s age and the molecular subtype.^3,9^ While Luminal A was more common in patients over 70,^9^ the aggressive TNBC subtype was more commonly diagnosed in those under 40.^3,9^ In this study, a similar trend was observed where, overall, Luminal A was the most prevalent breast cancer subtype in women aged 70 years and older (59.8%). In contrast, Luminal B was the most common subtype of breast cancer in women under 40 years (57.1%). The TNBC, on the other hand, was most frequently observed in the age group of 50–59 years. As supported by the literature, there are variations in pathological characteristics based on age, with more aggressive and hormone receptor-negative tumours typically occurring in younger patients.^3,9,12,20,34^

### Strengths and limitations

The study provided valuable information on the trend of breast cancer 10 years apart in SA concerning age distribution, tumour size, receptor status and molecular subtypes. Because of the doubled number of records in 2022 versus 2012, the authors had to compare two populations of different sizes. The introduction of a new PACS contributed to the lack of continuity. The inconsistent terminology in pathology reports and the absence of Ki-67 reporting in 2012 hindered direct comparisons. Incomplete data regarding receptor status and grade were accounted for as missing, which may have led to an underestimation of molecular subtype representations.

### Future applications

A cancer registry would help compare results within SA and globally. Although this study covered 10 years, future research could enable a more detailed comparative analysis for the 5 years before and after 2022, uncovering more accurate and comparable trend variations. While there may have been more women diagnosed in the 40–49 year age group in this study, this might not reflect the national trend, suggesting that more research will be necessary to determine whether this indicates a trend in the years following 2022. Future research may also benefit from incorporating the stage at diagnosis to understand better the factors influencing the aggressiveness of breast cancer.

## Conclusion

The average age of breast cancer diagnosis remained consistent for 2012 and 2022, suggesting that the demographic profile of breast cancer patients has remained stable. Reproductive and hormonal factors and lifestyle changes, however, could be contributing to the increasing prevalence of breast cancer in women aged 40–49 years. Newly diagnosed tumours in 2022 were significantly smaller, attributing to an increased breast cancer awareness, the DoH policy implementing CBE in local clinics and screening, which have facilitated earlier detection. Despite these trends, additional research is required on the age at first breast cancer presentation and its associated characteristics across more provincial healthcare facilities. This will assist in determining whether a national trend towards younger presentation is emerging. Such findings could help guide national screening and imaging guidelines for breast cancer and improve patient care.
